# Phylogenomics and phylogeography of *Menispermum* (Menispermaceae)

**DOI:** 10.3389/fpls.2023.1116300

**Published:** 2023-02-22

**Authors:** Shiqiang Song, Kenneth M. Cameron, Yuguo Wang, Shenyi Wang, Xinjie Jin, Faiza Hina, Zhaoping Yang, Pan Li

**Affiliations:** ^1^ College of Life Sciences and Technologies, Tarim University, Alar, China; ^2^ Laboratory of Systematic & Evolutionary Botany and Biodiversity, College of Life Sciences, Zhejiang University, Hangzhou, China; ^3^ Department of Botany, University of Wisconsin, Madison, WI, United States; ^4^ Ministry of Education Key Laboratory for Biodiversity Science and Ecological Engineering, Institute of Biodiversity Science, Fudan University, Shanghai, China; ^5^ College of Life and Environmental Science, Wenzhou University, Wenzhou, China

**Keywords:** disjunct distribution, Menispermaceae, plastome evolution, phylogeny, systematic

## Abstract

**Introduction:**

Phylogenomics have been widely used to resolve ambiguous and controversial evolutionary relationships among plant species and genera, and the identification of unique indels in plastomes may even help to understand the evolution of some plant families. *Menispermum* L. (Menispermaceae) consists of three species, *M. dauricum* DC., *M. canadense* L., and *M. mexicanum* Rose, which are disjuncly distributed among East Asia, Eastern North America and Mexico. Taxonomists continue to debate whether *M. mexicanum* is a distinct species, a variety of *M. dauricum*, or simply a synonym of *M. canadense*. To date, no molecular systematics studies have included this doubtful species in phylogenetic analyses.

**Methods:**

In this study, we examined phylogenomics and phylogeography of *Menispermum* across its entire range using 29 whole plastomes of Menispermaceae and 18 ITS1&ITS2 sequences of Menispermeae. We reconstructed interspecific relationships of *Menispermum* and explored plastome evolution in Menispermaceae, revealing several genomic hotspot regions for the family.

**Results and discussion:**

Phylogenetic and network analyses based on whole plastome and ITS1&ITS2 sequences show that *Menispermum* clusters into two clades with high support values, Clade A (*M. dauricum*) and Clade B (*M. canadense* + *M. mexicanum*). However, *M. mexicanum* is nested within *M. canadense* and, as a result, we support that *M. mexicanum* is a synonym of *M. canadense*. We also identified important molecular variations in the plastomes of Menispermaceae. Several indels and consequently premature terminations of genes occur in Menispermaceae. A total of 54 regions were identified as the most highly variable plastome regions, with nucleotide diversity (*Pi*) values > 0.05, including two coding genes (*mat*K, *ycf*1), four introns (*trn*K intron, *rpl16* intron, *rps*16 intron, *ndh*A intron), and 48 intergenic spacer (IGS) regions. Of these, four informative hotspot regions (*trn*H-*psb*A, *ndh*F-*rpl*32, *trn*K-*rps*16, and *trn*P-*psa*J) should be especially useful for future studies of phylogeny, phylogeography and conservation genetics of Menispermaceae.

## Introduction

The moonseed family (Menispermaceae, Ranunculales) includes approximately 72 genera and 526 species. It is most well known as a botanical source for the arrow poison curare (Krukoff and Moldenke, 1938.; Barbosa-Filho et al., 2000; Chen et al., 2013). Many systematic studies have focused on this family, from morphological taxonomic work (Miers, 1851; Prantl, 1888; Diels, 1910; Forman, 1986; Kessler, 1993) to molecular systematics based on plastid sequences (Ortiz et al., 2007; Hoot et al., 2009; Jacques et al., 2011; Wang et al., 2012; Wefferling et al., 2013; Lian et al., 2020), internal transcribed spacer (ITS) (Hong et al., 2001; Wang et al., 2007b), and the combination of morphological and molecular characteristics (Ortiz et al., 2016). The circumscription of tribes/clades within Menispermaceae has been modified several times since the first phylogenetic study using ITS was published (Hong et al., 2001), and ten tribes have been re-delimited in the last few years (Ortiz et al., 2016; Lian et al., 2020). The type genus of the family, *Menispermum* L., belongs to one of these tribes, Menispermeae DC., together with a second monotypic genus, *Sinomenium* Diels, represented by *S. acutum* (Thunb.) Rehder & E.H. Wilson (Ortiz et al., 2016; Lian et al., 2020).

**Figure 1:**
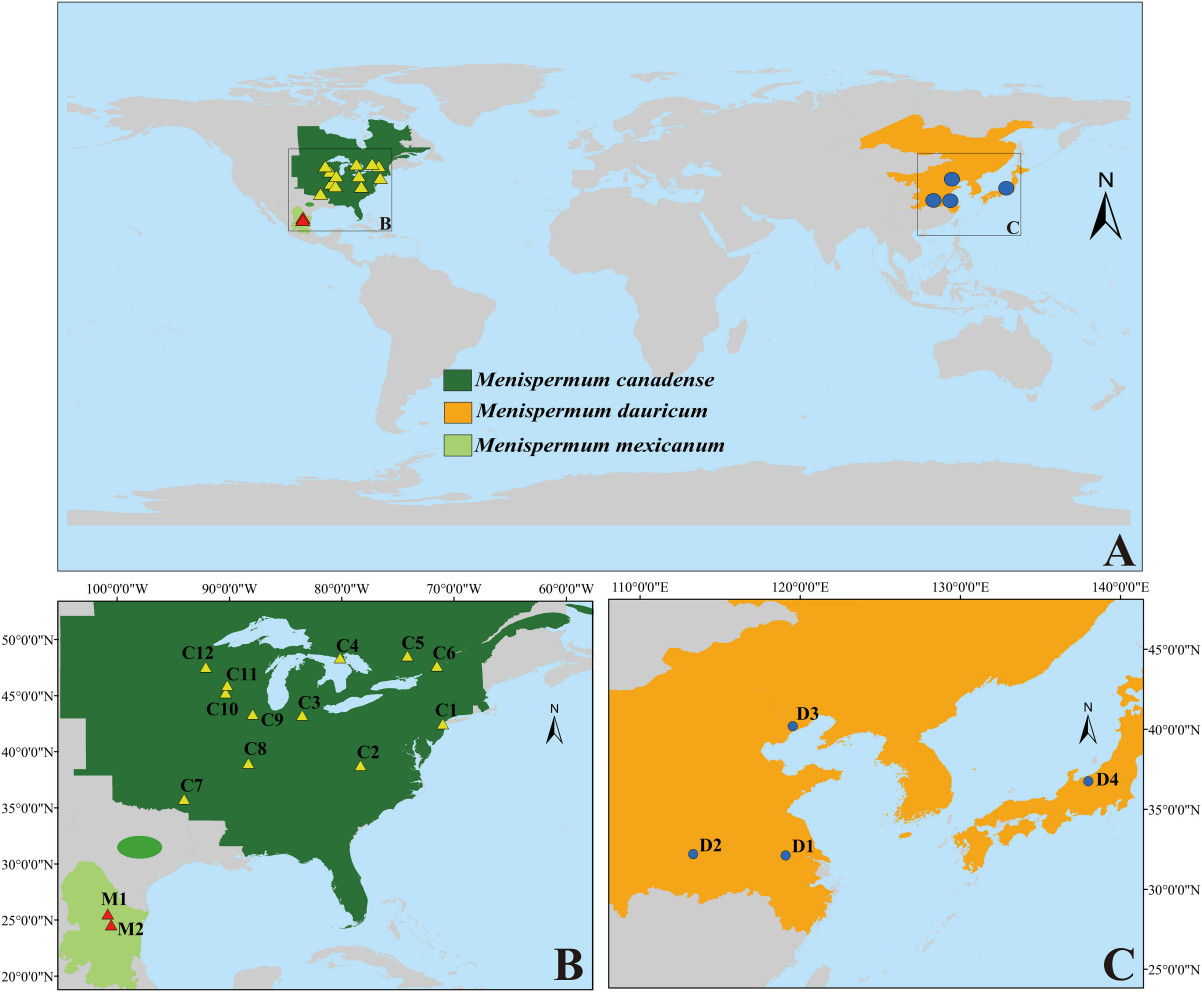
The distribution/sampling sites of *M. canadense* (dark green/yellow triangle), *M. dauricum* (orange/blue dot), and *M. mexicanum* (light green/red triangle). **(A)** world map; **(B)** eastern North America; **(C)** East Asia. Sample codes are the same as in **Table 1**.

*Menispermum*, a genus of deciduous climbing woody lianas, is disjuntcly distributed in East Asia, Eastern North America and Mexico (Xiang et al., 2000; Hina et al., 2020; **Figure 1**). Unlike most members of Menispermaceae, which are endemic to the tropics and subtropics, the ranges of *Menispermum* extend well into the northern temperate zone. For example, *M. dauricum* DC. (described in 1817) grows in central to northeastern China, southern Siberia, Korea, and Japan, growing amongst roadside vegetation and/or within open forests (Wu et al., 2008). *Menispermum canadense* L. (described in 1753) occurs in temperate eastern North America, growing in deciduous woods and thickets (Purrington and Horn, 1993; Morin, 1997). *Menispermum mexicanum* Rose (published in 1911) is considered to be a third species in the genus because of its larger drupes, glaucous lower leaves and disjunct distribution (northern Mexico) from *M. canadense*. Although the two species inhabit differ hemispheres, Kundu and Guha (1980) stated that *M. mexicanum* is most similar to *M. dauricum* and proposed that *M. mexicanum* is a variety of *M. dauricum*, based on detailed morphological and comparative anatomical studies. However, Calderón de Rzedowski (1999) proposed that *M. mexicanum* is a synonym of *M. canadense*. Despite its taxonomic uncertainty, *M. mexicanum* has never been sampled in any published molecular studies.

Today there are numerous molecular tools available to plant systematists, and analyses of whole plastomes have become widely used to resolve the circumscription of taxa at different levels of classification within angiosperms, including the families of early-diverging eudicots (Sun et al., 2016), the genera of Ranunculaceae (Liu et al., 2018b; Zhai et al., 2019), and even among species of *Aconitum* L. (Kong et al., 2017). Comparative plastome analyses also have helped to explore the structural variation of plastomes in angiosperm, such as gene indel (insertion/deletion) events, gene rearrangements, and/or inverted repeat expansion-contraction (Sun et al., 2016; Sun et al., 2018; He et al., 2019; Song et al., 2022). Plastome data can also be a good tool to analyze the phylogeographic patterns among plants (Sun et al., 2018; Demenou et al., 2020; Duan et al., 2020; Xiang et al., 2021). Due to characteristics of plastomes such as maternal inheritance, low to moderate evolutionary rate, their haploid nature which enhances genetic drift, plastome sequences may show a stronger phylogeographical pattern compared to nuclear DNA (Sugiura, 1992; Moore et al., 2010; Demenou et al., 2020; Zhang et al., 2021).

Here, we newly sequenced 19 individuals of Menispermeae, including twelve *M. canadense*, four *M. dauricum*, two *M. mexicanum* and one *Sinomenium acutum*. Together with ten publicly available plastomes (*Arcangelisia gusanlung* H.S. Lo, Wen et al., 2021; *Fibraurea recisa* Pierre, Zheng and Feng, 2022; *Pericampylus glaucus* (Lam.) Merr., Kang and Wang, 2019; *Stephania dielsiana* Y.C. Wu; *Stephania epigaea* H.S. Lo, Guan et al., 2022; *Stephania japonica* (Thunb.) Miers, Sun et al., 2016; two *Stephania tetrandra* S. Moore, Cao et al., 2020; *Sinomenium acutum*, Kim et al., 2020; *Tinospora sinensis* (Lour.) Merr.), we analyzed a matrix containing a total of 29 plastomes representing seven genera and twelve species. We also analyzed the matrix of ITS1&ITS2 for *Menispermum*. These results were used specifically to address the following questions: (1) how many species are there in *Menispermum*? and (2) do significant structural genomic changes appear across the plastomes of Menispermaceae?

## Materials and methods

### Plant material and DNA extraction

Fresh leaves from three *Menispermum* species and *Sinomenium acutum* were collected and then dried in silica-gel. In total twelve samples of *M. canadense*, four of *M. dauricum*, and one *Sinomenium acutum* were included. Voucher specimens were mostly deposited at the Herbarium of Zhejiang University (HZU), with the two *M. mexicanum* specimens (one of which is an isotype specimen) were sampled from the Gray Herbarium (GH) and Arnold Arboretum Herbarium (A) of Harvard University (**Table 1**). Total genomic DNA was extracted using DNA Plantzol Reagent (Invitrogen, Carlsbad, CA, United States) according to the manufacturer’s protocol. Agarose gel electrophoresis and an ultraviolet spectrophotometer (K5800, KAIAO, Beijing, China) were used to check the quality and quantity of genomic DNA, respectively.

**Table 1:** Collection locality and voucher information of Menispermeae samples newly sequenced in this study.

Species and sample code	Collection locality	Voucher information
*Menispermum canadense* (C1)	40 Old Mill Rd, Staten Island, NY, USA	*Pan Li* LP161702-1 (HZU)
*M. canadense* (C2)	Mindemoya, Manitoulin Island, ON, Canada	*Pan Li* LP150390-2 (HZU)
*M. canadense* (C3)	340 W Shoaff Rd, Huntertown, IN, USA	*Pan Li* LP185927-1 (HZU)
*M. canadense* (C4)	Mindemoya, Manitoulin Island, ON, Canada	*Pan Li* LP185840-2 (HZU)
*M. canadense* (C5)	V3MG+M5 Spotswood, ON, Canada	*Pan Li* LP185849-2 (HZU)
*M. canadense* (C6)	6150 County Rd 27, Williamstown, ON, Canada	*Pan Li* LP185879-2 (HZU)
*M. canadense* (C7)	Mena, Polk County, AR, USA	*Pan Li* LP162210-6 (HZU)
*M. canadense* (C8)	Jonesboro, Union County, IL, USA	*Pan Li* LP185636-1 (HZU)
*M. canadense* (C9)	Oglesby, LaSalle County, IL, USA	*Pan Li* LP162010-1 (HZU)
*M. canadense* (C10)	Bagley, Grant County, WI, USA	*Shenyi Wang* SY170138-1 (HZU)
*M. canadense* (C11)	Viroqua, Vernon County, WI, USA	*Shenyi Wang* SY180227-1 (HZU)
*M. canadense* (C12)	Hudson, St. Croix County, WI, USA	*Pan Li* LP162312-7 (HZU)
*M. dauricum* (D1)	Baohua Mountain, Baohua Village, Jurong City, Jiangsu Province, China	*Pan Li* LP161322-1 (HZU)
*M. dauricum* (D2)	Longwangchong Village, Wanhe Town, Sui County, Hubei Province, China	*Pan Li* LP161369-6 (HZU)
*M. dauricum* (D3)	Beigou, Shenmiao Village, Zushan Town, Qinhuangdao City, Hebei Province, China	*Pan Li* LP161437-1 (HZU)
*M. dauricum* (D4)	Kitajo, Nagano City, Nagano Prefecture, Japan	*Pan Li* LP162809-4 (HZU)
*M. mexicanum* (M1)	Sierra Madre above Monterey, Mexico	*C. G. Pringle* 10378 (isotype, GH)
*M. mexicanum* (M2)	Sierra Madre Oriental; waterway below Alamar, about 15 m. S. W. of Galeana, Mexico	*C. H. and M. T. Mueller* 626 (A)
*Sinomenium acutum* (FL2)	Qiyun Mountain, Qiyunshan Town, Xiuning County, Anhui Province, China	*Pan Li* LP196752 (HZU)

### Genome sequencing, assembly, and annotation

Short-inserts of 500-bp paired-ends were used to construct the libraries by Genomic DNA Sample Prep Kit (Illumina, San Diego, CA, United States). We used tags to index DNA from each species and pooled samples together for sequencing on a HiSeqTM 2500 platform at the Beijing Genomics Institute (BGI, Shenzhen, China) to obtain clean reads of each sample. Then, these reads were assembled into contigs or plastid sequences using GetOrganelle v 1.7.5.3 (Jin et al., 2020), and visualized in Bandage v 0.8.1 (Wick et al., 2015) to identify whether these contigs or plastid sequences were the whole plastome for each sample. Additionally, due to low sequencing depth of two *M. mexicanum* herbarium samples, only small size of contigs were obtained by GetOrganelle. To obtain longer plastid sequences, these short contigs were mapped to the reference *M. dauricum* (MH298220, Hina et al., 2018). Yet, there are still a lot of gaps. To obtain these gap-sequences, NOVOPlasty v 4.2 (Dierckxsens et al., 2017) was applied to assemble these sequences,with the neighboring protein-coding or tRNA genes in the plastome reference corresponding these gap sequences as seeds, and using the same plastome reference above. At last, these newly assembled gap-sequences were inserted into the above longer plastid sequences of *M*. *mexicanum* using software Geneious Prime^®^ 2021.2.2 (www.geneious.com) and we eventually generated their whole plastomes. Plastomes were annotated using ‘2544-plastome’ dataset of CPGAVAS2 (Shi et al., 2019), with default setting. Then, these whole plastomes were illustrated with the online tool OrganellarGenome DRAW v1.3.1 (Greiner et al., 2019) and deposited in GenBank (**Table 1**). We also downloaded ten additional whole plastomes of Menispermaceae from NCBI, i.e., *A. gusanlung* (MW829779), *F. recisa* (OK539642); *T. sinensis* (MN727386), *P. glaucus* (MN539265), *S. dielsiana* (MW1453970), *S. epigaea* (MZ678241), *S. japonica* (KU204903), *S. tetrandra* (MT849286, MT859132), *Sinomenium acutum* (MN626719) and reannotated these plastomes to perform a comparative plastome analysis of these Menispermaceae. The 18 ITS1&ITS2 were also assembled using GetOrganelle, but *M. mexicanum* (M2) were failed.

### Comparative plastome analyses

Altogether, by combining new and previously published plastomes (**Table 1**), a total of 12 plastomes representing 12 species of Menispermaceae were used to investigate the plastome evolution in this family. Interspecific variation was documented using mVISTA (Frazer et al., 2004) in Shuffle-LAGAN mode. Rearrangements of plastomes were checked by Mauve 2.3.0 (Darling et al., 2004). Gene and structure differences in the junctions of single copies and inverted repeat regions were visualized and compared through the software IRscope (Amiryousefi et al., 2018). The protein-coding sequences (CDS), intergenic spacer (IGS) and intron regions were extracted sequentially according to the criterion that alignment length > 200 bp and containing at least one mutation to estimate the nucleotide diversity (*Pi*) of plastid sequences of Menispermaceae. *Pi* value of each sequence was calculated in DNASP v 6.12.03 (Rozas et al., 2017).

### Phylogenetic and phylogeographic analyses

The phylogeny of Menispermaceae based on whole plastome/nrDNA was inferred using maximum likelihood (ML) and Bayesian inference (BI) methods *via* RAxML-HPC2 on XSEDE v 8.2.12 (Stamatakis, 2014) on CIPRES Science Gateway website (https://www.phylo.org) and MrBayes v 3.2.6 (Ronquist et al., 2012), respectively. For ML analysis, we set 1000 bootstrap replicates but used defaults for the other parameters. For BI analysis, we first evaluated the proper model of evolution (GTR+F+I+G4 for ptDNA, GTR+F+I for nrDNA) based on the Akaike Information Criterion (AIC) in ModelFinder (Kalyaanamoorthy et al., 2017), then ran two independent Markov chain Monte Carlo (MCMC) chains, with a set of 1,000,000 generations for each chain, and sampled one time for every 1,000 generations; the first 25% of the trees were discarded.

For phylogeographic analyses, we focused only on the 18 samples of *Menispermum*. The 18 plastomes were aligned with defaulted parameters by the plug-in of MAFFT in Geneious Prime^®^ 2021.2.2, and inferred the number of haplotypes (Nh) (excluding sites with gaps/missing data) in DNASP. The genealogical relationships of haplotypes were identified *via* TCS haplotype network using the software PopART v 1.7 (Leigh et al., 2015). The 17 ITS1&ITS2 sequences of *Menispermum* were analyzed using the same methods.

## Results

### Plastome features

**Figure 2:**
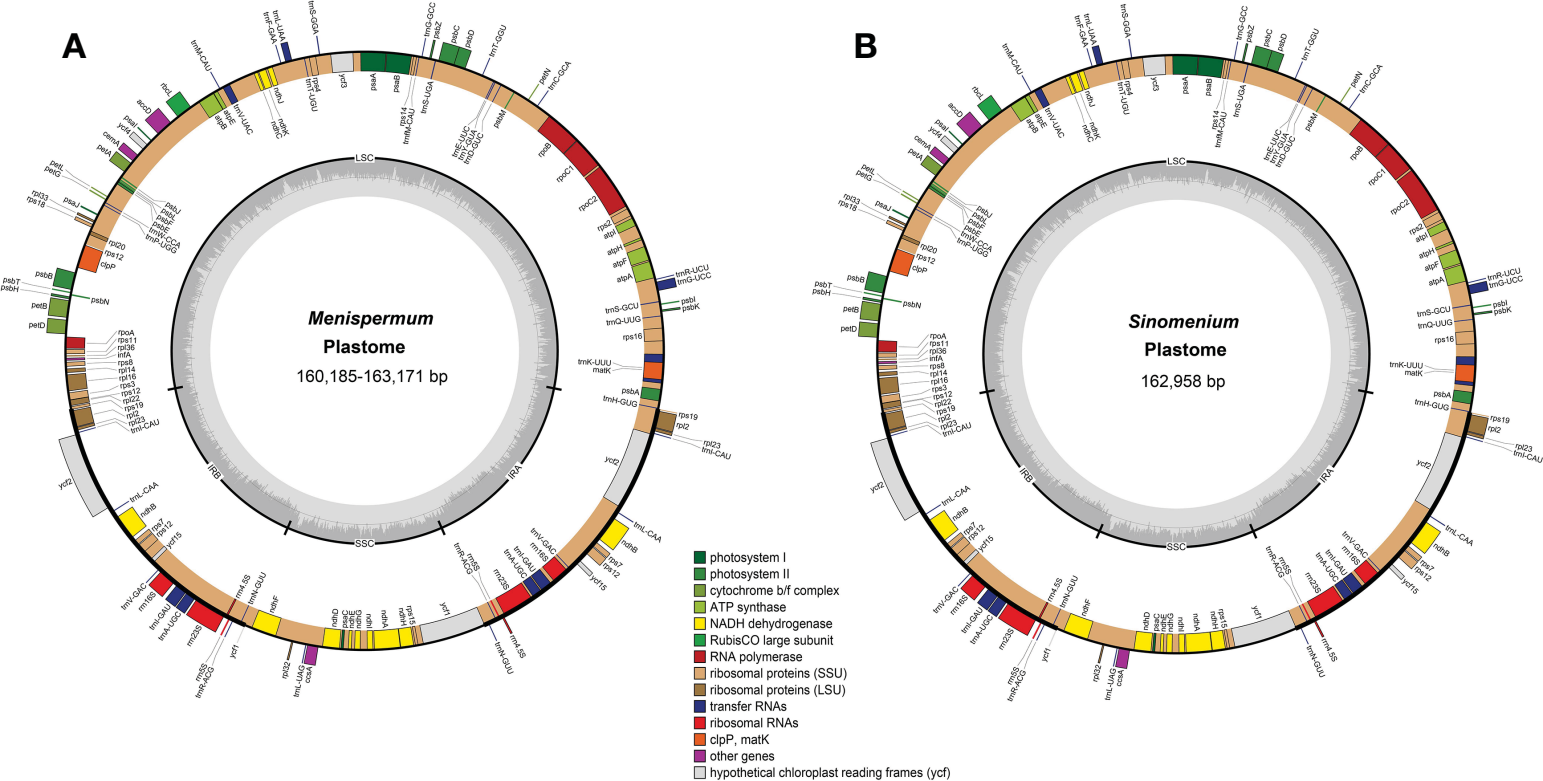
Representative plastome maps of the newly sequenced plastomes of Menispermaceae (19 in total). **(A)** *Menispermum*; **(B)** *Sinomenium*.

Total coverage of the 19 newly sequenced plastomes ranged from 9 × [*M. mexicanum* (M2)] to 536.9 × [*M. canadense* (C10)] (**Table 2**). These whole plastomes of Menispermaceae show a typical angiosperm quadripartite structure, including a pair of IR regions (IRa and IRb) and two single copy regions (LSC and SSC) (**Figure 2**). All the plastomes contain 114 unique genes, consisting of 80 CDS genes, 30 tRNA genes, and four rRNA genes (**Table S1**). The sizes of these newly assembled whole plastomes of Menispermeae range from 160,185 bp [*M. dauricum* (D4)] to 163,171 bp [*M. mexicanum* (M2)], and LSC, SSC and IR ranged from 89,380 bp in *M. dauricum* (D4) to 91,765 bp in *M. mexicanum* (M2), 20,781 bp in *M. dauricum* (D1) to 21,286 bp in *M. canadense* (C1), and 24,887 bp in *M. dauricum* (D1) to 25,064 bp in *M. dauricum* (D3), respectively (**Table 2**). The GC content of the 19 plastomes ranges from 37.7% to 38.4%, with the values in the IR regions (43.4–43.7%) being the greatest, followed by the LSC (35.8–36.6%) and SSC (32.1–33.4%).

**Table 2:** The basic characteristics of plastomes and ITS1&ITS2 sequences of Menispermaceae species.

Species	plastomes														ITS1/ITS2	
	Accession number	Av. cov. (×)	Length (bp)				GC cotent (%)				Gene number					
			Total	LSC	SSC	IR	Total	LSC	SSC	IR	Total	PCG	rRNA	tRNA	Accession number	Length
*A. gusanlung*	MW829779	–	162,509	91,449	20,852	25,104	37.8	35.9	32.6	43.5	134	87	8	37	–	–
*F. recisa*	OK539642	–	161,671	91,071	20,858	24,871	37.8	35.8	32.3	43.6	133	87	8	37	–	–
*T. sinensis*	MN727386	–	158,706	88,636	19,908	25,081	38.0	36.2	32.5	43.4	134	87	8	37	–	–
*P. glaucus*	MN539265	–	162,450	90,871	21,137	25,221	38.0	36.2	32.1	43.5	134	87	8	37	–	–
*S. dielsiana*	MW145397	–	156,843	87,538	19,631	24,837	38.4	36.6	33.4	43.6	134	87	8	37	–	–
*S. epigaea*	MZ678241	–	157,738	88,460	19,778	24,750	38.3	36.5	33.1	43.6	134	87	8	37	–	–
*S. japonica*	KU204903	–	157,719	88,583	20,346	24,395	38.2	36.4	32.9	43.7	134	87	8	37	–	–
*S. tetrandra*	MT849286	–	157,725	89,468	19,685	24,286	38.2	36.3	33.0	43.7	134	87	8	37	–	–
*S. tetrandra*	MT859132	–	159,974	90,539	20,735	24,350	37.8	35.8	32.4	43.7	134	87	8	37	–	–
*S. acutum*	MN626719	–	162,787	91,423	21,245	25,056	37.8	36.0	32.3	43.5	134	87	8	37	–	–
***S. acutum* (FL2)**	**OP271866**	134.1×	162,958	91,627	21,229	25,051	37.8	35.9	32.3	43.5	134	87	8	37	**OQ198602/OQ198621**	239/199
***M. canadense* (C1)**	**OP271873**	374.6×	163,095	91,687	21,286	25,061	37.8	36.0	32.3	43.5	134	87	8	37	**OQ198586/OQ198605**	240/199
***M. canadense* (C2)**	**OP271882**	491.7×	162,821	91,434	21,265	25,061	37.9	36.0	32.4	43.5	134	87	8	37	**OQ198594/OQ198613**	241/199
***M. canadense* (C3)**	**OP271880**	154.4×	162,819	91,433	21,264	25,061	37.9	36.0	32.4	43.5	134	87	8	37	**OQ198592/OQ198611**	240/199
***M. canadense* (C4)**	**OP980572**	292.2×	163,001	91,648	21,233	25,060	37.8	36.0	32.4	43.5	134	87	8	37	**OQ198595/OQ198614**	240/199
***M. canadense* (C5)**	**OP271883**	353.7×	163,003	91,635	21,248	25,060	37.8	36.0	32.4	43.5	134	87	8	37	**OQ198596/OQ198615**	241/199
***M. canadense* (C6)**	**OP271884**	449.8×	162,821	91,435	21,264	25,061	37.9	36.0	32.4	43.5	134	87	8	37	**OQ198597/OQ198616**	240/199
***M. canadense* (C7)**	**OP271876**	326.0×	162,999	91,634	21,245	25,060	37.8	36.0	32.4	43.5	134	87	8	37	**OQ198588/OQ198607**	240/199
***M. canadense* (C8)**	**OP271879**	296.7×	163,045	91,677	21,248	25,060	37.8	36.0	32.4	43.5	134	87	8	37	**OQ198591/OQ198610**	240/199
***M. canadense* (C9)**	**OP271875**	434.6×	160,303	91,677	21,233	25,060	37.8	36.0	32.4	43.5	134	87	8	37	**OQ198587/OQ198606**	240/199
***M. canadense* (C10)**	**OP271878**	536.9×	163,001	91,648	21,233	25,060	37.8	36.0	32.4	43.5	134	87	8	37	**OQ198590/OQ198609**	241/199
***M. canadense* (C11)**	**OP271881**	292.4×	163,004	91,637	21,247	25,060	37.8	36.0	32.4	43.5	134	87	8	37	**OQ198593/OQ198612**	240/199
***M. canadense* (C12)**	**OP271877**	407.3×	161,237	89,871	21,246	25,060	37.9	36.2	32.4	43.5	134	87	8	37	**OQ198589/OQ198608**	241/199
***M. dauricum* (D1)**	**OP271867**	483.7×	160,629	90,000	20,781	24,887	38.0	36.1	32.9	43.6	133	87	8	37	**OQ198598/OQ198617**	236/199
***M. dauricum* (D2)**	**OP271868**	424.7×	162,861	91,507	21,230	25,062	37.8	36.0	32.4	43.5	134	87	8	37	**OQ198599/OQ198618**	236/199
***M. dauricum* (D3)**	**OP271869**	470.8×	162,845	91,551	21,166	25,064	37.8	36.0	32.5	43.5	134	87	8	37	**OQ198600/OQ198619**	236/199
***M. dauricum* (D4)**	**OP271870**	246.6×	160,185	89,380	20,797	25,004	38.1	36.3	33.0	43.5	134	87	8	37	**OQ198601/OQ198620**	236/199
***M. mexicanum* (M1)**	**OP271871**	13.3×	163,055	91,650	21,285	25,060	37.8	36.0	32.3	43.5	134	87	8	37	**OQ198585/OQ198604**	240/159
***M. mexicanum* (M2)**	**OP271872**	9.0×	163,171	91,765	21,284	25,061	37.8	36.0	32.4	43.5	134	87	8	37	–	–

### Phylogenetic and the phylogeographic analyses

**Figure 3:**
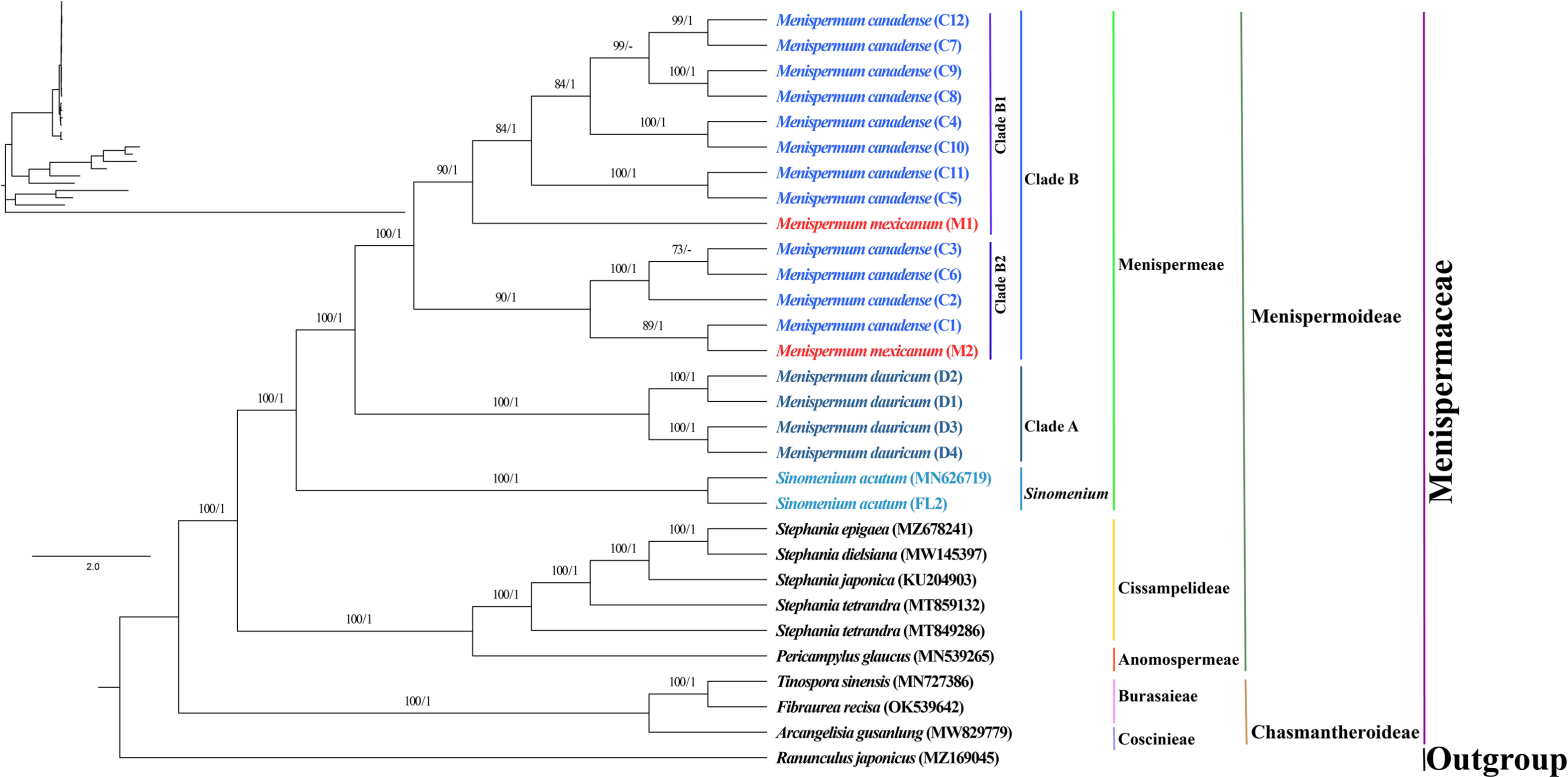
Maximum likelihood (ML) and Bayesian inference (BI) phylogeny based on 29 complete plastomes of Menispermaceae, with *Ranunculus* as outgroup. Numbers at each node represent ML bootstrap support (BS) and BI posterior probability (PP) values, respectively. Hyphens indicate the nodes not found in the strict consensus BI tree. The phylogram on the upper left shows the relative branch lengths. Sample codes are the same as in **Table 1**.

**Figure 4:**
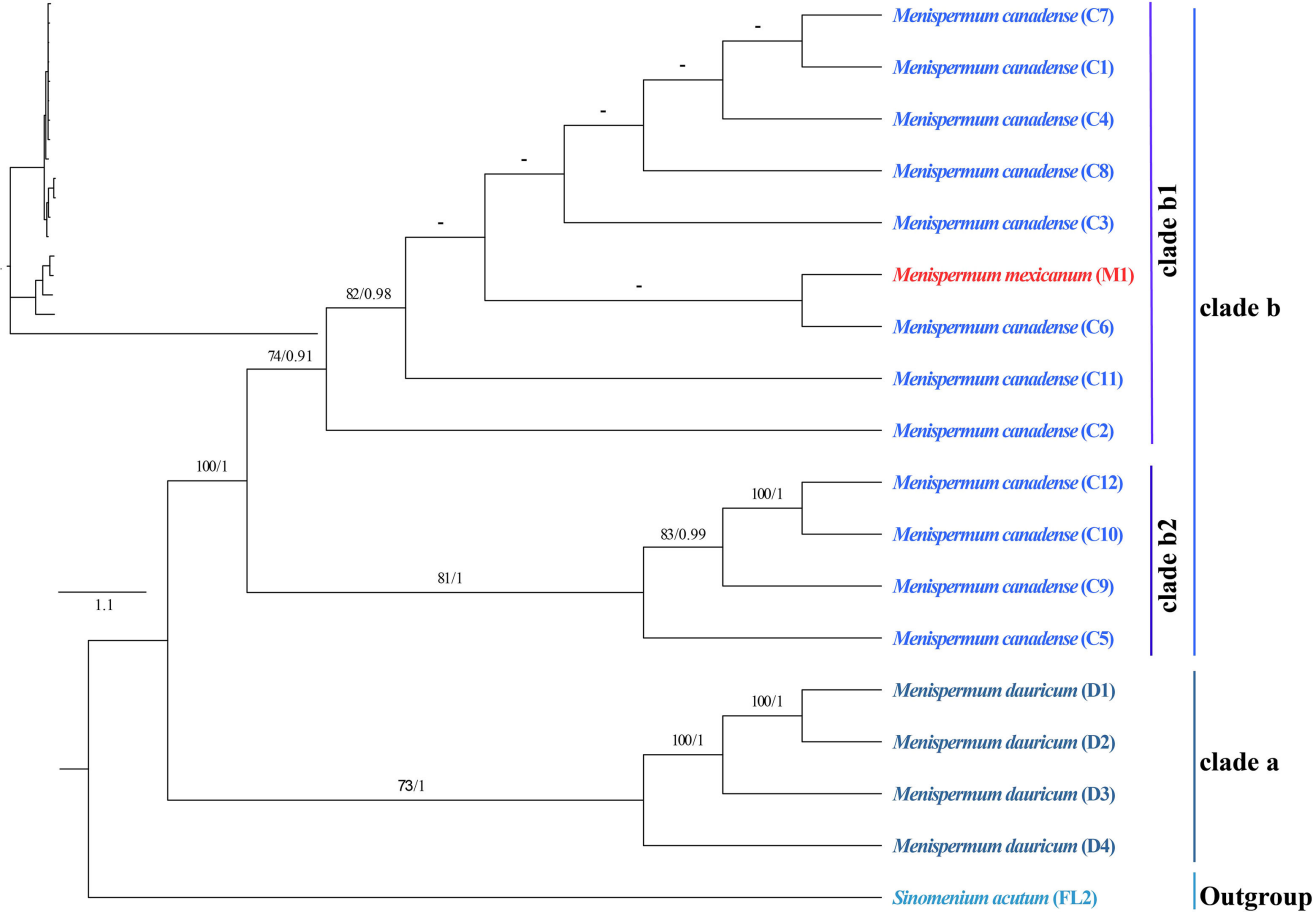
Maximum likelihood (ML) and Bayesian inference (BI) phylogeny based on 17 ITS1&ITS2 sequences of *Menispermum*, with *Sinomenium* as outgroup. Numbers at each node represent ML bootstrap support (BS) and BI posterior probability (PP) values, respectively. Hyphens indicate the nodes not found in the strict consensus BI tree. The phylogram on the upper left shows the relative branch lengths. Sample codes are the same as in **Table 1**.

The phylogenetic trees we generated using ML and BI methods resulted in similar topologies with high support (**Figure 3**). The Coscinieae + Burasaieae clade is the first diverging lineage of the family. This is followed by the clade of Anomospermeae + Cissampelideae as sister to tribe Menispermeae, within which *Sinomenium* is sister to the species of *Menispermum*. *Menispermum dauricum* (Clade A) is monophyletic and sister to the Clade B of *M. canadense + M. mexicanum.* Clade B is divided into two highly supported clades, Clade B1 (BS = 90, PP = 1) and Clade B2 (BS = 90, PP = 1) each of which contains accessions of both *M. mexicanum* and *M. canadense*, rendering them not monophyletic. The ML and BI trees based on the ITS1&ITS2 sequences show similar topology with the plastid tree, i.e., *Menispermum dauricum* (clade a) is sister to clade b that consists of *M. canadense* and *M. mexicanum* (**Figure 4**). In clade b, *M. mexicanum* (M1) was nested in clade b1 which is sister to clade b2.

**Figure 5:**
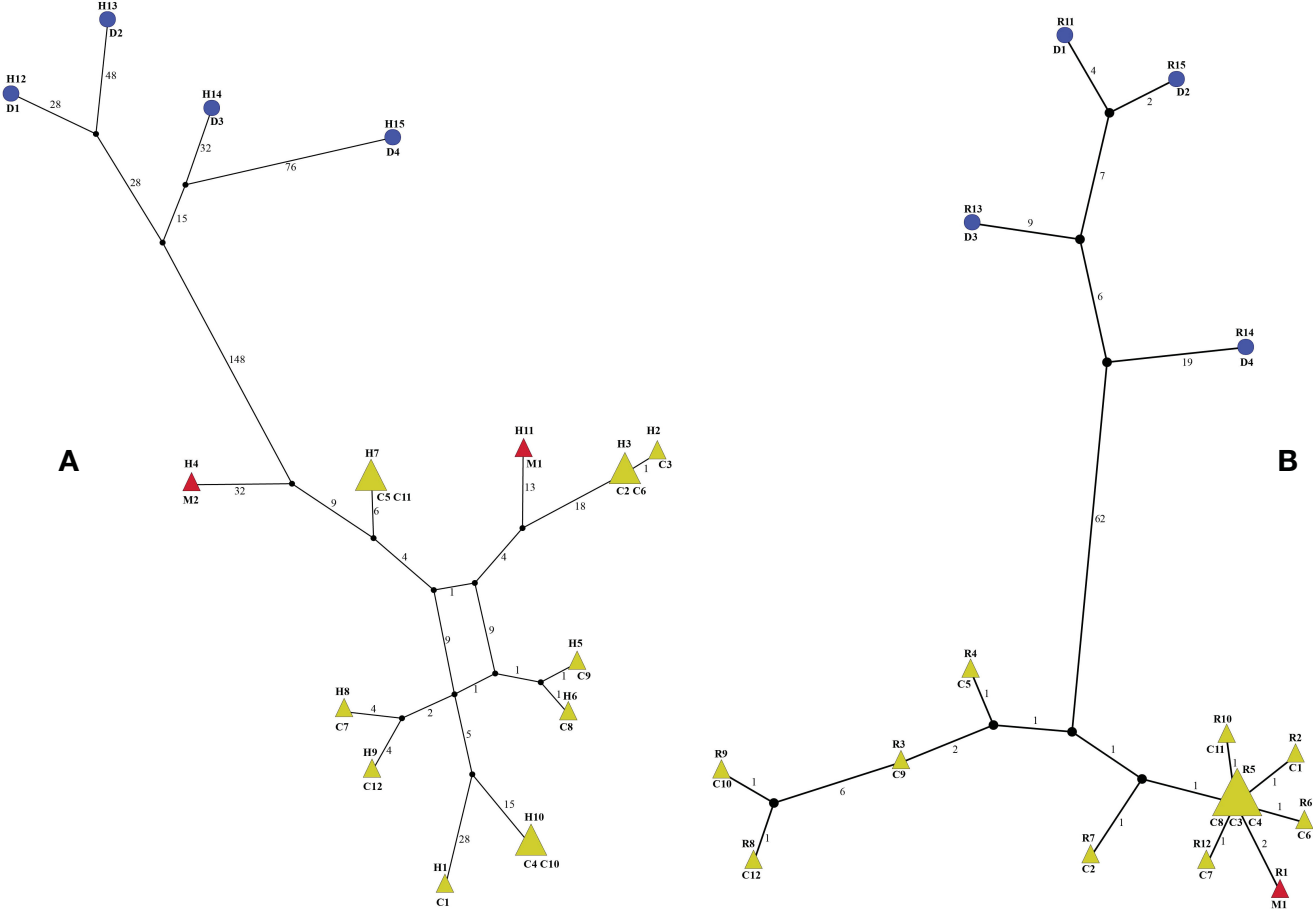
Haplotype **(A)** and ribotype **(B)** networks of *Menispermum*. C, D, and M represent *M. canadense*, *M. dauricum*, and *M. mexicanum*, respectively. Sample codes are the same as in **Table 1**.

The 18 whole plastomes of *Menispermum* were identified to have 15 haplotypes (**Figure 5A**). All of these are unique except that haplotypes H3, H7 and H10 are shared in two samples (**Figure 5A**). The 17 ITS1&ITS2 sequences of *Menispermum* were identified to have 15 ribotypes (**Figure 5B**). All of these are unique except that ribotype R5 is shared by three samples (C3, C4, C8) (**Figure 5B**). The haplotypes and ribotypes cluster significantly into two lineages, i.e. *M. dauricum* and *M. canadense* + *M. mexicanum*. Yet, the haplotypes and ribotype of *M. mexicanum* sampled are all embedded within *M. canadense.*

### Comparative plastome analyses

**Figure 6:**
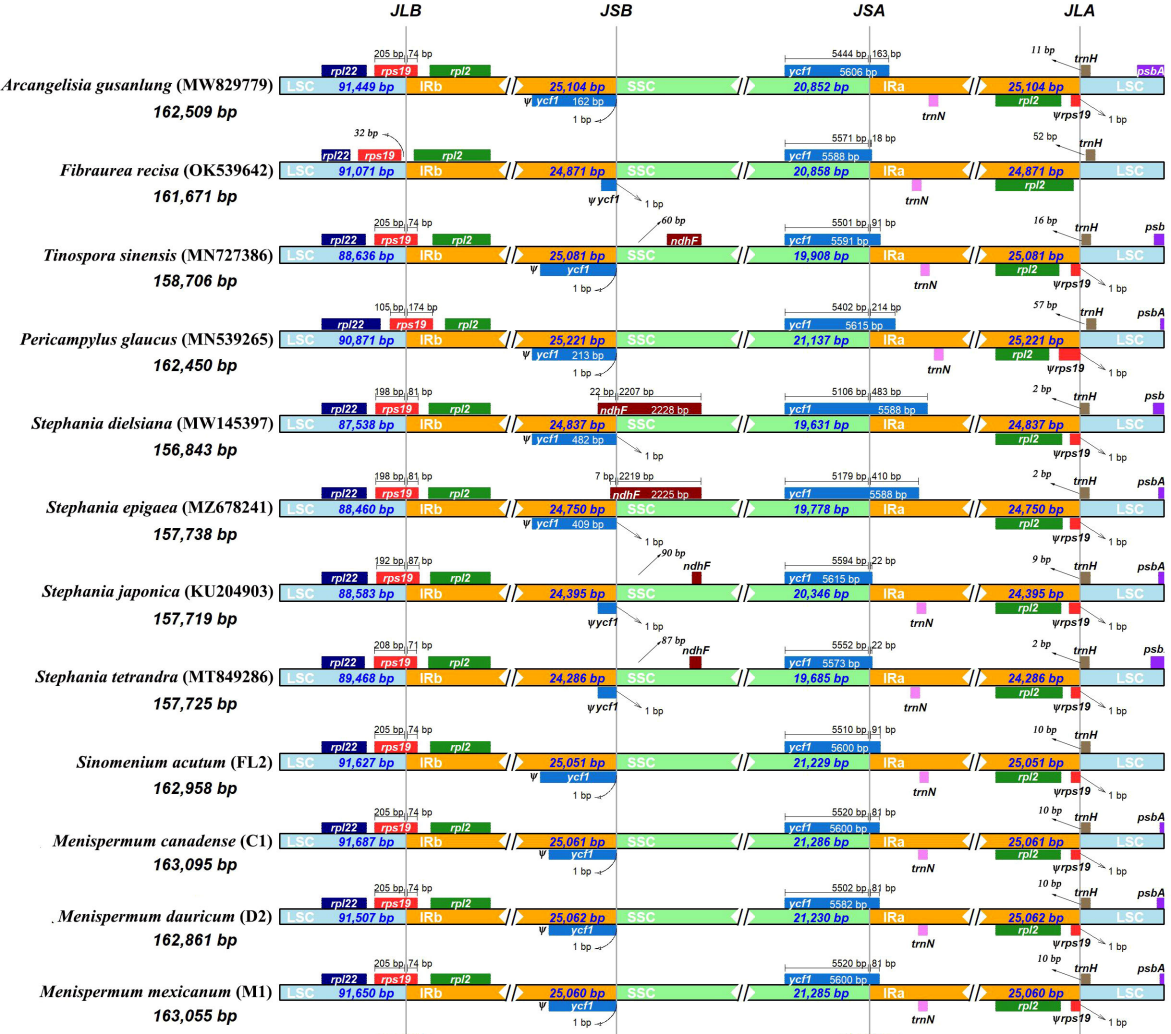
Comparison of the LSC/IRb/SSC/IRa junctions among the 12 complete plastomes of Menispermaceae. Sample codes are the same as in **Table 1**.

Due to minor variations in each species, we performed a comparative plastome analysis of Menispermaceae using only 12 plastomes representing 12 species. The *Stephania* species have the shortest plastomes (~157 kb), whereas species of Menispermeae have the longest (~163 kb). Global visualization using mVISTA and MAUVE showed that all of these 12 plastomes of Menispermaceae have a consistent gene order, except that the *rpo*C2 gene in the plastome of *T. sinensis* is inverted (**Figures S1**, **S2**). The *rps*19 gene of *F. recisa* is located in LSC, with a 32 bp-distance away from the junction of LSC and IRb (JLB), whereas the other eleven species have their *rps*19 located at the boundary of LSC/IRb (JLB) and the genes copied 71–174 bp sequences (*ψrps*19) in IRa (**Figure 6**). The junction of SSC/IRa (JSA) in the all 12 plastomes are located in *ycf1* gene (5574 bp- 5615bp), and the gene copied different length of sequences (*ψycf*1) in IRb, which ranged from 18 bp in *F. recisa* to 483 bp in *S. dielsiana* (**Figure 6**). Especially, only two species (*S. dielsiana* and *S. epigaea*) have their *ndh*F gene across the SSC/IRB boundary (JSB).

We also calculated the length of all genes across 29 plastomes in Menispermaceae. Of all 114 genes, 26 genes varied in length, and only minor variations (less than 42 bp) were documented among 18 genes, i.e. *mat*K, *atp*F, *rpo*C1, *rpo*B, *ndh*K, *atp*E, *acc*D, *rps*18, *rpl*20, *pet*D, *rpo*A, *inf*A, *rpl*23, *ndh*F, *rpl*32, *ccs*A, *rps*15, *ycf*1 (**Table S2**). The *rpo*C2 gene in the plastome of *T. sinensis* occurred a base replace from “T” to “C” in the locus of 2137 bp, which results in a premature termination of that gene, about 2,000 bp shorter than the other plastomes. The *ycf*2 gene in all *Stephania* plastomes has an approximately 417 bp deletion at locus 1787/1788 bp, compared to the plastomes from the other genera. In the plastome of *P. glaucus*, the *ycf*15 gene is about 200 bp shorter than the other 28 Menispermaceae plastomes because of a 10-bp insertion of “TATTCTATTA” in the locus of 211 bp, resulting in a premature termination. The sequences in the second extron of *rps*16 gene in *S. dielsiana* and *S. epigaea*, are significantly different from the other 27 plastomes, with “AGAATAAAA” and “AGAATAAAT” replacing “GT” or “AT” in the loci of 103-111 bp, which results in the *rps*16 gene being almost halved in the two plastomes, compared to the remaining plastomes.

**Figure 7:**
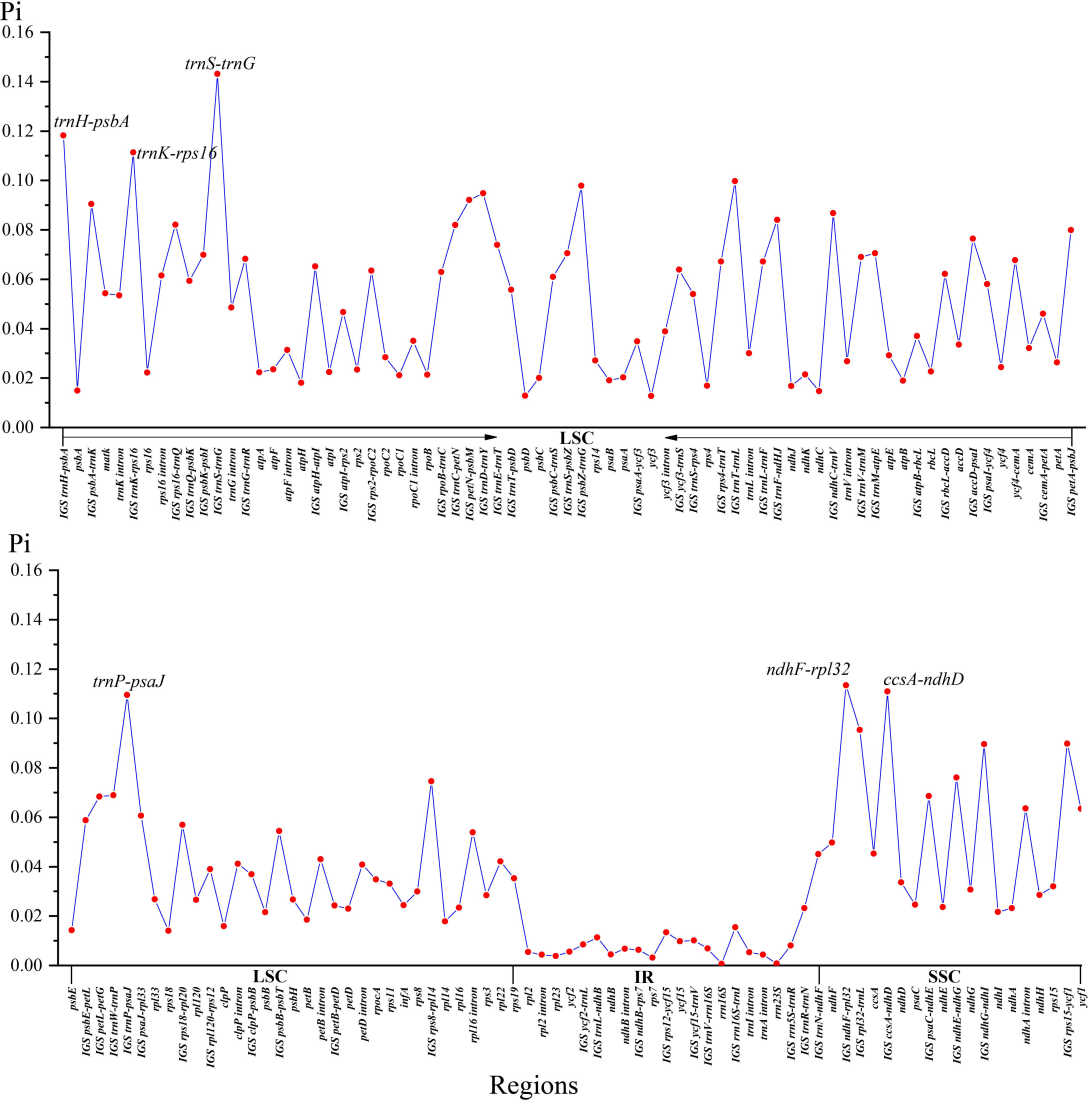
Nucleotide diversity (*Pi*) values of 12 Menispermaceae plastome sequences.

Two single-copy (SSC, LSC) regions exhibit a higher level of sequence variation than the IR regions (**Figure 2**). In the alignment of the 12 plastomes of Menispermaceae, a total of 147 regions were extracted to calculate the value of nucleotide diversity (*Pi* values). They included 65 intergenic spacer (IGS) regions, 63 protein-coding (CDS) regions, 17 intron regions (of CDS/tRNA genes), and two rRNA gene (**Figure 7**). A total of 54 regions with *Pi* more than 0.05 in LSC or SSC were revealed (**Figure 7**). The *Pi* in CDSs and intron regions showed significantly lower than those in IGS regions. These CDSs ranged from 0.00317 (*rps*7) to 0.06347 (*ycf*1), only *mat*K and *ycf*1 genes showed high values more than 0.05. For the intron regions, *Pi* ranged from 0.00436 (*trn*A intron) to 0.06359 (*ndh*A intron), four introns (*trn*K, *rpl16*, *rps*16 and *ndh*A) gene showed high diversity (*Pi* > 0.05). Besides, for the 65 IGS regions, the value of *Pi* ranged from 0.00631 (*ndh*B-*rps*7) to 0.14317 (*trn*S*-trn*G), 48 regions showed high diversity, and six showed remarkably high diversity (*Pi* > 0.1; i.e., *trn*S-*trn*G, *trn*H-*psb*A, *ndh*F-*rpl*32, *trn*K-*rps*16, *ccs*A-*ndh*D, *trn*P-*psa*J; see **Figure 5**). The sizes and *Pi* values of the six hotspot regions are shown in **Table S3**, and corresponding phylogenetic trees based on each region are shown in **Figure S3**.

## Discussion

### Phylogenetic, phylogeographic and taxonomic inferences

Our molecular phylogeny of Menispermaceae (represented by five tribes, seven genera, twelve species and 29 accessions) fully resolves the relationships among these taxa with robust support and bifurcates into two major clades that correspond to the subfamilies Chasmantheroideae and Menispermoideae (**Figure 3**). Within the latter subfamily, the tribe Menispermeae is sister to the (Cissampelideae + Anomospermeae) clade, consistent with previous studies based on plastid sequences (*rbc*L, *atp*B, *mat*K, *ndh*F and *trn*L-F) and/or combined plastid+nuclear ITS region (Ortiz et al., 2007; Ortiz et al., 2016; Wang et al., 2007b; Wang et al., 2012; Wefferling et al., 2013; Ortiz et al., 2016) (**Figure 3**).

Within tribe Menispermeae, *Sinomenium* is sister to *Menispermum* with maximum support (BS = 100, PP = 1, **Figure 3**), consistent to the previous results based on plastid and/or combined rDNA sequences (Hoot et al., 2009; Jacques et al., 2011; Wang et al., 2012; Wefferling et al., 2013; Ortiz et al., 2016; Lian et al., 2020). The species *M. dauricum* (Clade A) from East Asia is monophyletic, and the two accessions from eastern (D1) and central China (D2) clustered into a subclade, whereas the remaining two from northern China (D3) and Japan (D4) clustered into another subclade (**Figure 1** and **Table 1**). A similar lineage divergence between north and south has been found in unrelated plants such as *Saxifraga* sect. *Irregulares* (Zhang et al., 2019) indicating that this pattern is not random. Clade B (**Figure 3**) contains the accessions of *M. canadense* from the eastern United States and Canada plus two accessions of *M. mexicanum* from northern Mexico, which are not sister to each other. Based on the holotype specimen (U. S. National Herbarium no. 462662) collected by Dr. C. G. Pringle in the Sierra Madre, Mexico on July 9^th^, 1907, Rose (1911) proposed a new species, *M. mexicanum*, because of its leaves not being glaucous beneath, larger drupes, and a much more southern range than *M. canadense*. However, Kundu and Guha (1980) considered *M. mexicanum* to be a variety of Asian *M. dauricum* based on their detailed morphological and comparative anatomical studies. Our study is the first molecular systematics study that includes all three species. Both accessions of *M. mexicanum* are from different herbarium specimens collected in Mexico (they cannot be misidentifications of *M. canadense*) and one of them *M. mexicanum* (M1) was sampled directly from a historical isotype specimen. These two accessions of *M. mexicanum* are nested within *M. canadense* in both phylogenetic tree (**Figures 3**, **4**) and TCS diagram (**Figure 5**), rendering neither species monophyletic. Hence, we here propose that *M. mexicanum* is merely a geographic disjunct population and a synonym of *M. canadense.* This result supports the taxonomic treatment published by Calderón de Rzedowski (1999).

### Comparative plastome analyses

All of the 19 newly sequenced plastomes of Menispermeae in our study contain 114 unique genes (**Table S1** and **Figures S1**, **S2**), consistent with the eleven previously reported Menispermaceae plastomes (Sun et al., 2016; Hina et al., 2018; Kang and Wang, 2019; Cao et al., 2020; Kim et al., 2020; Wen et al., 2021; Guan et al., 2022; Zheng and Feng, 2022). However, these plastomes have two more genes (*ycf*15, *rpl*32) than the tribe Anemoneae, and one more gene (*ycf*15) than the genera *Anemoclema* (Franch.) W.T. Wang (Jiang et al., 2017), *Archiclematis* Tamura, *Clematis* L., and *Naravelia* DC. of Ranunculaceae, the sister family of Menispermaceae (Kim et al., 2004; Liu et al., 2018a).

The *rpo*C2 gene in *T. sinensis* contains an inversion and significant truncation of about 2 kb (**Table S2** and **Figure S2**), which is possibly due to the base substitution of “T/C” in the locus 2,137 bp and resulting in the premature termination of that gene. The pseudogene ψ*ycf*1 of *S. dielsiana* and *S. epigaea*, which may have resulted from an IR expansion, is ~200 bp longer than that of the ten other Menispermaceae species (Song et al., 2022). The *rps*19 gene of *F. recisa* is located in the LSC region, resulting in the fact that ψ*rps*19 does not appear in IRa. This phenomenon also has been observed in other plant families, such as some species of *Aconitum* (Ranunculaceae), *Dicorynia paraensis* Benth. (Fabaceae), and at least three species of *Oxalis* L. (Oxalidaceae) (Kong et al., 2017; Bai et al., 2021; Li et al., 2021).

The plastome length of our twelve focus species shows significant differences (**Figure 6**), ranging from 156,843 bp (*Stephania dielsiana*) to 163,095 bp [*M. canadense* (C1)], which is mainly ascribed to insertions/deletions (indels) in the intergenic spacer regions. Yet, some gene length differences are significant between tribes or genera, and even among species. For example, an ~417 bp deletion within the *ycf*2 gene is present in all *Stephania* plastomes. A 12 bp deletion in the *atp*F gene, which may be a molecular synapomorphy of the tribe Menispermeae, occurs only in the genera *Sinomenium* and *Menispermum*. For *Menispermum*, the *ndh*F gene in four *M. dauricum* plastomes has a 6 bp insertion compared with those from *M. canadense* and *M. mexicanum*. Indels and consequently premature termination of various genes were also found in the plastome evolution of Menispermeae, such as an about 200 bp truncation of *P. glaucus* caused by the 10-bp insertion of “TATTCTATTA”. In brief, indels and premature termination events of genes are usual phenomenon of plastome evolution in angiosperms and we have documented them within Menispermaceae as well (Wang et al., 2007a; Fu et al., 2017; Wang et al., 2020; Song et al., 2022).

### Highly variable regions of Menispermaceae plastomes

Several plastid gene and spacer sequences (*atp*B, *mat*K, *ndh*F, *rbc*L, and *trn*L-F) have been used to resolve the backbone phylogeny of Menispermaceae (Ortiz et al., 2007; Jacques et al., 2008; Hoot et al., 2009; Jacques et al., 2011; Wang et al., 2012; Wefferling et al., 2013; Ortiz et al., 2016; Lian et al., 2020). However, only 63 of 72 genera and fewer than 150 of 526 species were sampled in those previous studies (Hong et al., 2001; Ortiz et al., 2007; Wang et al., 2007b; Hoot et al., 2009; Jacques et al., 2011; Wang et al., 2012; Wefferling et al., 2013; Lian et al., 2020), suggesting that additional sampling of both taxa and data might be needed to get a full picture of evolutionary relationships within the family. In this study, of the six newly proposed hotspot regions, with nucleotide diversity (Pi) values > 0.1 just four loci (*trn*H-*psb*A, *ndh*F-*rpl*32, *trn*K-*rps*16 & *trn*P-*psa*J) are adequate to recover the monophyly of all genera and species that are represented by more than one individual (**Table S3**). This is encouraging and suggest that these markers will be useful candidate DNA barcodes for further studies on phylogeny and phylogeography of Menispermaceae (Barbosa-Filho et al., 2000; Singh and Bedi, 2016; He et al., 2018; Hina et al., 2020; Hao et al., 2022).

## Data availability statement

The data presented in the study are deposited in the NCBI repository, accession number OP271866-OP271873, OP271875-OP271884, OP980572, OQ198585-OQ198602, OQ198604-OQ198621..

## Author contributions

PL and ZY designed this study. PL and SW collected plant materials. SS and ZY assembled and analyzed the data, and prepared the figures and tables. ZY, SS, and PL wrote the original draft, KC, YW, and XJ modified the manuscript. All authors contributed to the article and approved the submitted version.

## Supplementary material

Supplementary Figure 1 Comparison of the 12 plastomes of Menispermaceae analyzed in this study using mVISTA, with *Ranunculus japonicus* (MZ169045) as a reference.

Supplementary Figure 2 MAUVE alignment of the 12 Menispermaceae plastomes analyzed in this study, with *Arcangelisia gusanlung* as a reference.

Supplementary Figure 3 Maximum likelihood (ML) trees based on each of the 6 hotspot regions (Pi > 0.1). **(A)** *trn*S-*trn*G; **(B)** *trn*H-*psb*A; **(C)** *ndh*F-*rpl*32; **(D)** *trn*K-*rps*16; **(E)** *ccs*A-*ndh*D; **(F)** *trn*P-*psa*J. Numbers listed at each node represents bootstrap support (BS) values. The hyphen indicates BS < 50%.
