# Supplementary figures and images for: Phylogenomics and phylogeography of Menispermum (Menispermaceae)
Source: Front Plant Sci. 2023 Feb 22;14:1116300. doi: 10.3389/fpls.2023.1116300 (PMC9992823; doi:10.3389/fpls.2023.1116300)

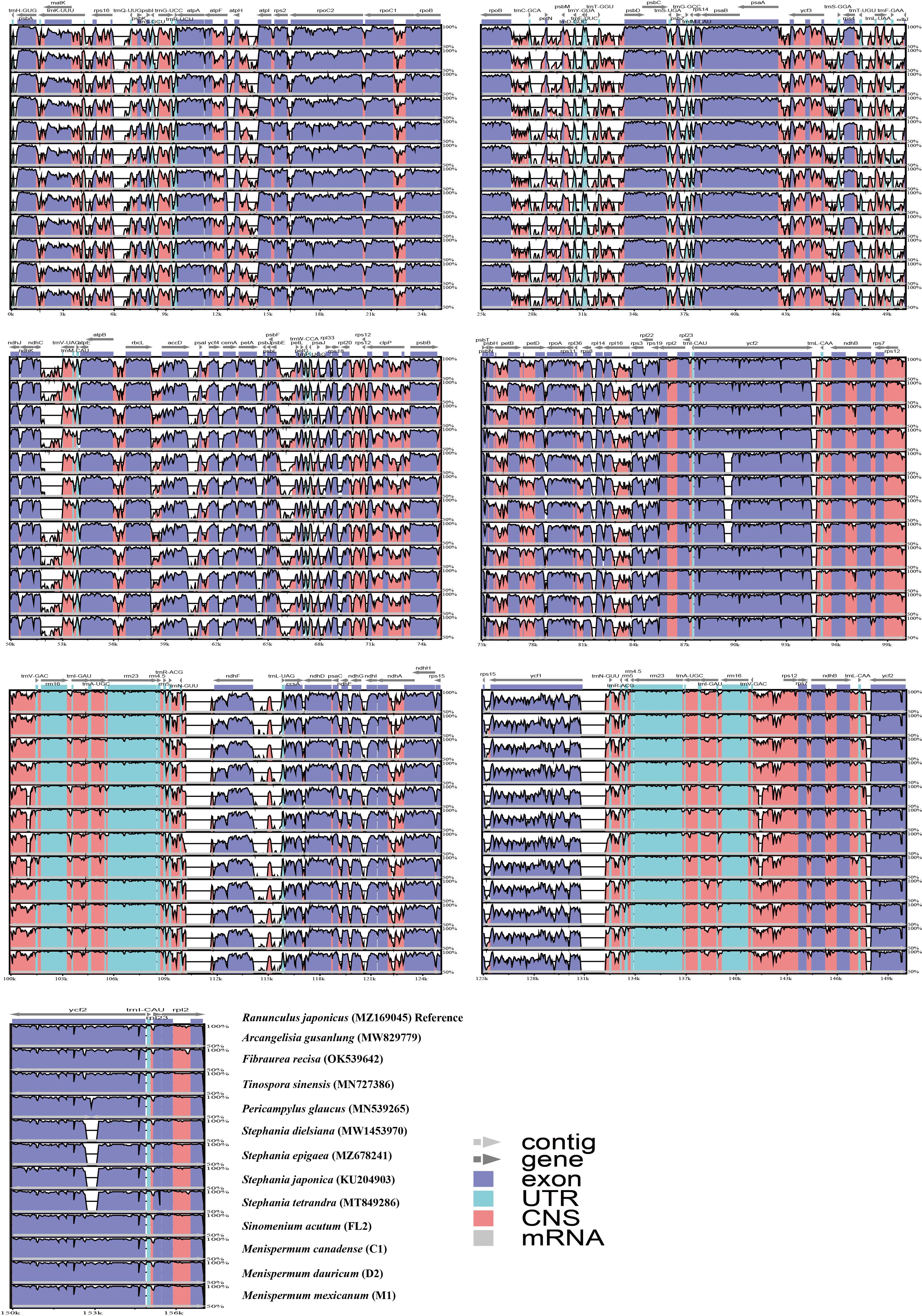

Supplement: Supplementary Figure 1 — Comparison of the 12 plastomes of Menispermaceae analyzed in this study using mVISTA, with Ranunculus japonicus (MZ169045) as a reference. [file Image_1.jpg]

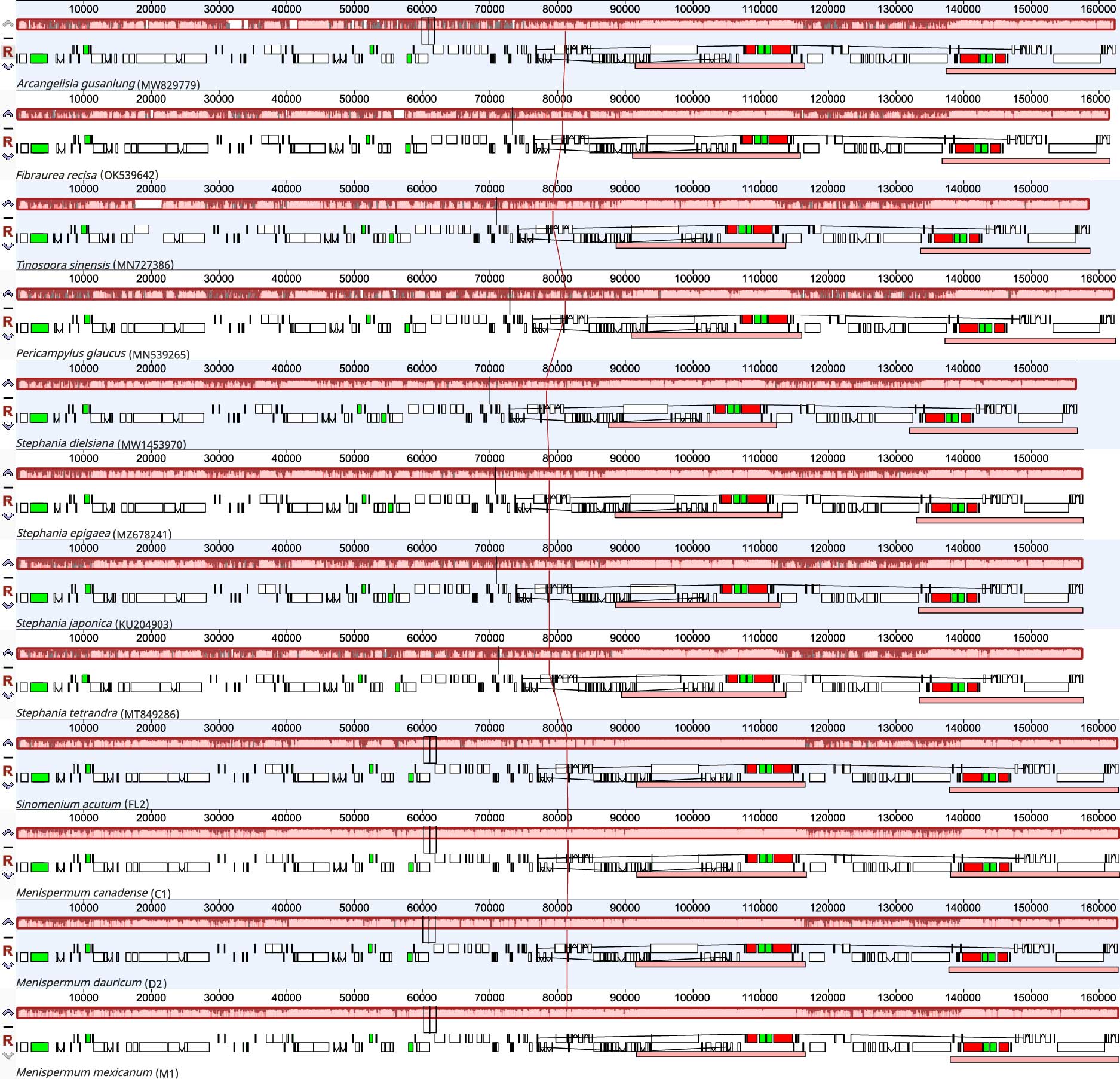

Supplement: Supplementary Figure 2 — MAUVE alignment of the 12 Menispermaceae plastomes analyzed in this study, with Arcangelisia gusanlung as a reference. [file Image_2.jpg]

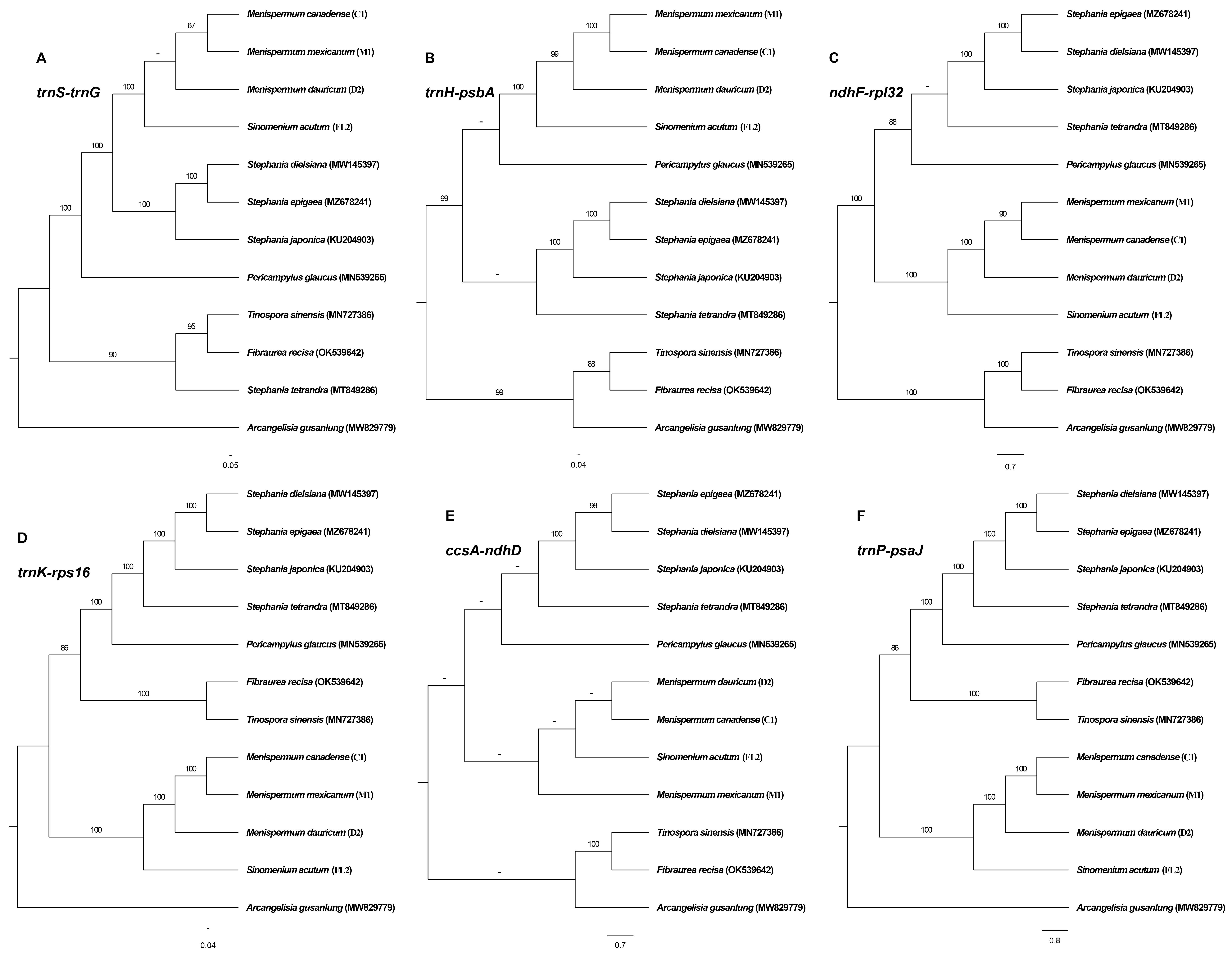

Supplement: Supplementary Figure 3 — Maximum likelihood (ML) trees based on each of the 6 hotspot regions (Pi > 0.1). (A) trnS-trnG; (B) trnH-psbA; (C) ndhF-rpl32; (D) trnK-rps16; (E) ccsA-ndhD; (F) trnP-psaJ. Numbers listed at each node represents bootstrap support (BS) values. The hyphen indicates BS < 50%. [file Image_3.jpg]
