# Supplementary material for: Phylogenomics and phylogeography of Menispermum (Menispermaceae)
Source: Front Plant Sci. 2023 Feb 22;14:1116300. doi: 10.3389/fpls.2023.1116300 (PMC9992823; doi:10.3389/fpls.2023.1116300)
Supplement: Supplementary file 4 [file Table_1.docx]

**Table S1. List of genes in the 19 plastomes newly sequenced in this study.**

| Gene category | Groups of genes | Name of genes | | | |
| --- | --- | --- | --- | --- | --- |
| Self-replication | Transfer RNA genes | *trn*A-UGC^a*^  *trnF-*GAA  *trnH-*GUG  *trnL-*CAA^a^  *trnN-*GUU^a^  *trnR-*UCU  *trnT-*GGU  *trnW-*CCA | *trn*C*-*GCA  *trnfM*-CAU  *trnI-*CAU^a^  *trnL-*UAA^*^  *trnP-*UGG  *trnS-*GCU  *trnT-*UGU  *trnY-*GUA | *trn*D*-*GUC  *trnG-*GCC^*^  *trnI-*GAU^a*^  *trnL-*UAG  *trnQ-*UUG  *trnS-*GGA  *trnV-*GAC^a^ | *trn*E*-*UUC  *trnG-*UCC  *trnK-*UUU^*^  *trnM-*CAU  *trnR-*ACG^a^  *trnS-*UGA  *trnV-*UAC^*^ |
|  | Small subunit of ribosome | *rps*2  *rps8*  *rps*15 | *rps*3  *rps*11  *rps*16^*^ | *rps*4  *rps*12^a,b*^  *rps*18 | *rps*7^a^  *rps*14  *rps*19^a^ |
|  | Ribosomal RNA genes | *rrn*4.5^a^ | *rrn*5^a^ | *rrn*16^a^ | *rrn*23^a^ |
|  | Large subunit of ribosome | *rpl*2^a^  *rpl*22  *rpl*36 | *rpl*14  *rpl*23^a^ | *rpl*16^*^  *rpl*32 | *rpl*20  *rpl*33 |
|  | RNA polymerase subunits | *rpo*A | *rpo*B | *rpo*C1^*^ | *rpo*C2 |
| Photosynthesis | Subunits of photosystem I | *psa*A  *psa*J | *psa*B  *ycf*3^**^ | *psa*C | *psa*I |
|  | Subunits of photosystem II | *psb*A  *psb*E  *psb*J  *psb*N | *psb*B  *psb*F  *psb*K  *psb*T | *psb*C  *psb*H  *psb*L  *psb*Z | *psb*D  *psb*I  *psb*M |
|  | Subunits of cytochrome | *pet*A  *pet*L | *pet*B^*^  *pet*N | *pet*D^*^ | *pet*G |
|  | Subunits of ATP synthase | *atp*A  *atp*H | *atp*B  *atp*I | *atp*E | *atp*F^*^ |
|  | Large subunit of Rubisco | *rbc*L |  |  |  |
|  | Subunits of NADH  Dehydrogenase | *ndh*A^*^  *ndh*E  *ndh*I | *ndh*B^a*^  *ndh*F  *ndh*J | *ndh*C  *ndh*G  *ndh*K | *ndh*D  *ndh*H |
| Other genes | Translational initiation factor | *inf*A |  |  |  |
|  | Maturase | *mat*K |  |  |  |
|  | Envelope membrane protein | *cem*A |  |  |  |
|  | Subunit of acetyl-CoA | *acc*D |  |  |  |
|  | C-type cytochrome  synthesis gene | *ccs*A |  |  |  |
|  | Protease | *clp*P^**^ |  |  |  |
| Unknown function | Conserved open reading frames | *ycf*1^a^ (part) | *ycf*2^a^ | *ycf*4 | *ycf15*^a^ |

^a^ Two gene copies in IRs; ^b^ gene divided into two independent transcription units; one and two asterisks indicate one- and two-intron containing genes, respectively.
