# Supplementary material for: Phylogenomics and phylogeography of Menispermum (Menispermaceae)
Source: Front Plant Sci. 2023 Feb 22;14:1116300. doi: 10.3389/fpls.2023.1116300 (PMC9992823; doi:10.3389/fpls.2023.1116300)
Supplement: Supplementary file 6 [file Table_3.docx]

**Table S3.** Sizes and *Pi* values of 6 hotspot regions (*Pi* > 0.1).

| Regions | Number of sites | Eta (Number of mutations) | *Pi* |
| --- | --- | --- | --- |
| IGS *trn*S*-trn*G | 1134 | 240 | 0.14317 |
| IGS *trn*H*-psb*A | 765 | 125 | 0.11819 |
| IGS *ndh*F*-rpl*32 | 1643 | 149 | 0.11340 |
| IGS *trn*K*-rps*16 | 1265 | 163 | 0.11138 |
| IGS *ccs*A*-ndh*D | 384 | 86 | 0.11088 |
| IGS *trn*P*-psa*J | 1074 | 194 | 0.10950 |
